# Psychotropic substance use among medical residents: prevalence and characteristics

**DOI:** 10.1192/j.eurpsy.2024.833

**Published:** 2024-08-27

**Authors:** H. Ktari, M. Moalla, N. Smaoui, I. Gassara, R. Feki, S. Omri, L. Zouari, M. maalej bouali, M. Maalej, J. Ben Thabet, N. Charfi

**Affiliations:** Psychiatry C, Hedi Chaker Hospital of Sfax, Sfax, Tunisia

## Abstract

**Introduction:**

Psychotropic substance use among medical residents represents a critical concern due to its potential impact on patient care and practitioner well-being. This topic looks into the prevalence and characteristics of psychotropic substance use, including prescription medications and illicit drugs, among individuals pursuing medical residency. Understanding the scope of the problem and its distinctive features is essential for developing targeted interventions and support mechanisms within the medical community.

**Objectives:**

To assess psychotropic substance use among medical residents, describe its characteristics and determine the prevalence of problematic use.

**Methods:**

We conducted a cross-sectional and descriptive studyamong Tunisian medical residents over a three-month period (August - September 2022) using an online survey. Different specialties and levels of residency were included. An online self-questionnaire was used including a data collection form and the DAST-10 (Drug Abuse Screening Test) scale. The data was analyzed using the SPSS 20th version software

**Results:**

Among the 80 residents in our study, 19 (23.8%) reported psychotropic substance use, and 12 (15%) reportedthat they had misused psychotropic drugs at least once in their lives (without a prescription and/or with a prescription but not following the instructions). The most commonly psychotropic drugs used were benzodiazepines, followed by amphetamines, analgesics, anesthetics, and pregabalin (28%, 16%, 12%, 12%, 12%, respectively).

Consumption was regular for 41.7% of those who reported psychotropic drugs misuse. The initiation of psychotropic use followed a desire to experiment various substances (41.7%), a medical prescription (33.3%), or their availability due to medical practice (25%). Self-medication and recreational use were the most common reasons for use (41.7% each). Furthermore, 31.6% of consumers revealed a problematic substance use as assessed by the DAST-10 scale.

**Conclusions:**

Our study showed a concerning prevalence of psychotropic substance use among medical residents, benzodiazepines being the most prevalent. Notably, 15% acknowledged misuse and a significant proportion displayed problematic substance use. These results highlight the potential health risks and the importance of addressing this issue within the medical community.

**Disclosure of Interest:**

None Declared

